# Phosphorylation of PFKFB4 by PIM2 promotes anaerobic glycolysis and cell proliferation in endometriosis

**DOI:** 10.1038/s41419-022-05241-6

**Published:** 2022-09-15

**Authors:** Chao Lu, Pengyun Qiao, Ruihai Fu, Yadi Wang, Jiayi Lu, Xi Ling, Lu Liu, Yujun Sun, Chune Ren, Zhenhai Yu

**Affiliations:** Department of Reproductive Medicine, Affiliated Hospital of Weifang Medical University, Weifang, Shandong Province P. R. China

**Keywords:** Phosphorylation, Phosphorylation

## Abstract

Endometriosis (EM) is one of the vanquished wonted causes of chronic pelvic sting in women and is closely associated with infertility. The long-term, complex, systemic, and post-treatment recurrence of EM wreaks havoc on women’s quality of life. Extensive metabolic reprogramming (aerobic glycolysis, glucose overweening intake, and high lactate production) and cancer-like changes have been found in EM, which bears striking similarities to tumorigenesis. The key glycolysis regulator PFKFB4 is overexpressed in EM. However, the mechanism of PFKFB4 in EM remains unknown. We found that PFKFB4 was upregulated and was closely related to the progression of EM. We identified focus PIM2 as a new pioneering adjoin protein of PFKFB4. Vigorous biochemical methods were used to confirm that PIM2 phosphorylated site Thr140 of PFKFB4. PIM2 also could enhance PFKFB4 protein expression through the ubiquitin–proteasome pathway. Moreover, PIM2 expression was really corresponding prevalent with PFKFB4 in endometriosis in vivo. Importantly, phosphorylation of PFKFB4 on Thr140 by PIM2 promoted EM glycolysis and cell growth. Our study demonstrates that PIM2 mediates PFKFB4 Thr140 phosphorylation thus regulating glycolysis and EM progression. We illustrated a new mechanism that PIM2 simulated a central upstream partnership in the regulation of PFKFB4, and reveal a novel means of PIM2-PFKFB4 setting EM growth. Our research provided new theoretical support for further clarifying the reprogramming of EM glucose metabolism, and provided new clues for exploring non-contraceptive treatments for EM.

## Introduction

Endometriosis (EM) is a complex chronic inflammatory disease affecting ~176 million women worldwide [1]. EM also is one of the vanquished wonted causes of chronic pelvic sting in women and is closely associated with infertility [2–4]. Its medical history can range from teenage to menopause, and the onset can involve various tissues even including the central nervous system [5–8]. The long-term, complex, systemic, and post-treatment recurrence of EM wreaks havoc on women’s quality of life [9, 10]. However, its pathogenesis is still unclear. Elucidating the pathogenesis is the premise to ensure effective diagnosis and precise treatment of EM.

6-Phosphofructose-2-kinase/fructose-2,6-bisphosphatase 4 (PFKFB4), a root regulator of glycolysis, plays a banderole regulatory role in the process of glycolysis [11]. It could participate in the synthesis and degradation of fructose-2,6-bisphosphate. Its kinase domain is necessary for the stimulation of glycolysis and cell growth which plays an important role in maintaining abnormal cell proliferation [12]. PFKFB4 can convert more glucose to NAPDH and 5-ribonucleic acid through the PPP pathway, thereby supporting cellular resistance to oxidative stress and promoting lipid and nucleotide synthesis [13]. Silencing PFKFB4 expression leads to PPP damage and reduces the production of NAPDH which results in increased levels of F-2,6-BP in glucose-dependent cells. This brought more glucose into the glycolytic flux and increased cellularity apoptosis [14]. Furthermore, PFKFB4 plays a foremost regulatory affair in glucose metabolism, cell proliferation, and cell cycle [11, 15]. Meanwhile, most EM carry somatic mutations which are known as cancer-related mutations [16]. Our previous preliminary experiments find that PFKFB4 is abnormally highly expressed in EM tissues, but the mechanism of its regulation in EM progression is still not well understood and needs further study.

Proviral insertion in murine lymphomas 2 (PIM2) belongs to the proto-oncogene family of serine/threonine kinases and is highly conserved during biological evolution [17]. PIM2 is generally expressed in ovarian cancer, endometrial cancer, breast cancer, and other reproductive system tumors abnormally and at high levels [18–20]. It can regulate biological processes such as cell metabolism and proliferation through phosphorylation modification, and promote tumorigenesis [21]. PIM2 could protect cancer cells from apoptotic signal regulation and evade immune attacks then promote tumor progression [22]. Studies have shown abnormal and high-level expression of PIM2 in endometrial biopsy ectopic tissue, and its abnormal level is in relation to the clinical stage of EM [23]. However, the specific mechanism by which PIM2 regulates EM progression through metabolic reprogramming is still unclear, and its underlying molecular mechanism requires further discussion.

Here, we identified that PIM2 can bind to PFKFB4 and specifically recognize the conserved RXXpT/S sequence of PFKFB4. PIM2 could change PFKFB4 phosphorylation level, promote its stable expression, and further promote EM glycolysis and cell proliferation. Therefore, we put forward a scientific hypothesis that phosphorylation of PFKFB4 results in EM glycolysis and development via PIM2. The development of this project will provide new theoretical support for further clarifying the reprogramming of EM glucose metabolism, and provide new clues for exploring non-contraceptive treatments for EM. Therefore, the implementation of this project has important theoretical exploration significance and practical value.

## Materials and methods

### Cell culture

Endometriosis epithelial cells 11Z were established by Prof. Anna Strazinski-Powitz [24]. Stromal cell ESC was established by Dr. Krikum [25]. HEK293T cells were purchased from the American Type Culture Collection (ATCC). Endometriosis cells were cultured in the F12/DMEM medium (Corning) while HEK293T cells were in the DEME medium (Corning). Primary mouse embryonic fibroblast was isolated as previously described and suspended in DMEM medium. All cells were cult cultured at 37 C in 5% CO_2_ (v/v). All cell lines were passaged less than 15 times. Mycoplasma detection on cells every 2 months.

### Plasmids and transfection

The cDNA of genes (PFKFB4, PIM1, PIM2, PIM3) were cloned into HA, Flag, GFP, His, or GST. Construction of related mutant plasmids by overlapping PCR technology. The ShPFKFB4, ShPIM2, and ShNC plasmids were constructed by the GenePharma company. The oligonucleotide of ShPFKFB4 was 5’-GCGCAGCTCTTAGGTGTTCAC-3’, The oligonucleotide of ShPIM2 was 5’-CTCGAAGTCGCACTGCTAT-3’, The oligonucleotide of ShNC was 5’-TTCTCCGAACGGTCACGT-3’. Plasmid transfection was performed according to the instructions. The transfection reagent used is lipofectamine 2000 (Invitrogen) [26].

### Antibodies

Site-specific antibodies against specific peptide sites TRERRAT(p)IFNFGE of PFKFB4 was generated by Shanghai Genomic Inc. Anti-DDDDK tag Rabbit (ab205606, 1:3000), Flag tag Mouse (ab18230, 1:1000), HA Tag Mouse (ab1424, 1:2000), HA tag Rabbit (ab236632, 1:1000) were purchased from Abcam. IgG (30000-0-AP, 1:500 and B900620, 1:500), were purchased from Abcam. PFKFB4(SAB1300132,1:2000), PIM2 Mouse (SAB1407095, 1:3000) were purchased from Sigma.

### Immunoprecipitation and western blotting

Cells were transfected in different experiments for different purposes. The cells were treated with 300 μl protein lysate. 50 μl protein supernatant was reserved as input. The remaining protein supernatant was incubated overnight at 4 °C with magnetic beads containing specific antibodies. Centrifuge at 900 × *g* for 90 s, the supernatant was removed, and the mixed magnetic beads were retained. The mixed magnetic beads were washed with ice-cold washing buffer more than 4 times, and remove the supernatant to retain the magnetic beads. The beads were denatured by an SDS loading buffer containing dithiothreitol [27]. Western Blot was used to analyze the samples as described previously [28].

### GST pull-down assay

The GST pull-down assays using GST or GST-PFKFB4 were as described previously [29].

### Confocal immunofluorescence microscopy

Cells were transfected for different experimental purposes. Cells in the logarithmic growth phase were selected and plated on the cell slides, and cultured overnight in a 5% CO_2_ 37 °C incubator. When cell density reached 50–70% before the experiment, 4% paraformaldehyde solution was used to fix the cells. After washing the cells with 1XPBS 3 times, 5% BSA blocking solution was added standing at room temperature for 1 h. Choice consummate antibodies were incubated overnight at 4 °C. TRITC red light or FITC green fluorescently labeled were incubated for 1 h at room temperature in the dark. After washing with 1XPBS, the slides were mounted with an anti-fluorescence decay mounting medium containing DAPI, and then placed under a laser confocal microscope for observation and photographing [28].

### Wound healing assay, clone formation, and cell proliferation analysis

The wound healing, clone formation, and cell proliferation analysis were performed as described previously [28, 30].

### Measurements of glucose uptake, lactate production

Cells were transfected according to different experimental purposes. When the cell confluency exceeded 90%, fresh medium was replaced for 12 h. The supernatant medium was collected and Measurements of glucose uptake (Sigma, GAGO20), and lactate production (Bio Vision, catalog K627) were determined according to the manufacturer’s instructions [28, 31].

### Tissue samples and endometriosis mouse model

Thirty cases of EM tissues and 30 cases of normal uterine tissue were obtained from the Affiliated Hospital of Weifang Medical College. The patients signed informed consent. All patients were premenopausal and received no hormonal or anti-coagulant treatment prior to the surgery. The diagnosis of endometriosis was established through laparoscopy and histological confirmation. As controls, endometrial tissue samples were obtained from cycling women, age- and menstrual phase-matched (in frequency) with those endometrioses who underwent hysterectomy with uterine leiomyoma or grade II–III cervical intraepithelial neoplasia (CIN), and without clinical indication or history of endometriosis or adenomyosis [32, 33]. The tissue sampled in our study was approved by the Human Assurance Committee of Affiliated Hospital of Weifang Medical University (Ethics number 2021YX028). Our analysis adhered to the avowal of Helsinki.

All mice experimental procedures were in accordance with the Health Guide for the Care and Use of Laboratory Animals and approved by the Animal Experimentation Ethical Committee of Weifang Medical University (Ethics number 2021SDL141). 21 8-week-old C57BL/6 female mice were purchased from Jinan Experimental Animal Breeding Company (Permit Number 20140007). PIM2 knockout (PIM2−/−) mice were generated from Cyagen. The matching forward strand of the gene was gRNA1 (GGGACAGACTTTACTTCTTTAGG) and gRNA2 (TTTGGTGCCAGGCTCCCCACTGG). Mice were randomly assigned for the following experiments. No blinding was carried out for animal experiments. No blinding was carried out for animal experiments.

Endometriosis mouse model: The donor mouse were injected intramuscularly with 100 μg/kg estradiol benzoate (Shenzhou pharmaceutical, Zhejiang, China) 3 times a week. The donor mice were then sacrificed and the uteruses were cut into very small segments in warm sterile saline. The fragments were mixed together and injected intraperitoneally into the recipient mouse. Then the recipient mouse was injected intramuscularly with 100 μg/kg estradiol benzoate 3 times. Recipient mice were euthanized 1 month later and endometriotic lesions were collected for subsequent experimental analysis [34].

### Immunohistochemistry (IHC)

Tissue samples were harvested, embedded, and fixed. Tissue wax blocks were dewaxed with xylene, and protein antigens were repaired using an antigen retrieval solution. 5% BSA blocking solution was added standing at room temperature for 30 min. Selected antibodies were incubated overnight at 4 °C. Tissues were treated with polymer augmenters A and B after washing with PBS. DAB was used for color development, and hematoxylin was used to stain nuclei. Tissues were dehydrated with different concentrations of alcohol, cleared with xylene, and mounted with neutral gum [20, 34].

### Statistical analysis

SPSS and Graphpad Prism 7 software were used to analyze and graph. Experiments were continued at least 3 times with a standard deviation of the mean (mean ± s.d.). Two-tailed Student *t-*tests were performed. Pearson significance direction was used to analyze the topic between two variables. ns was not significant, *p < 0.05, **p < 0.01.

## Results

### PFKFB4 is positively related to the progression of EM

**Fig. 1:**
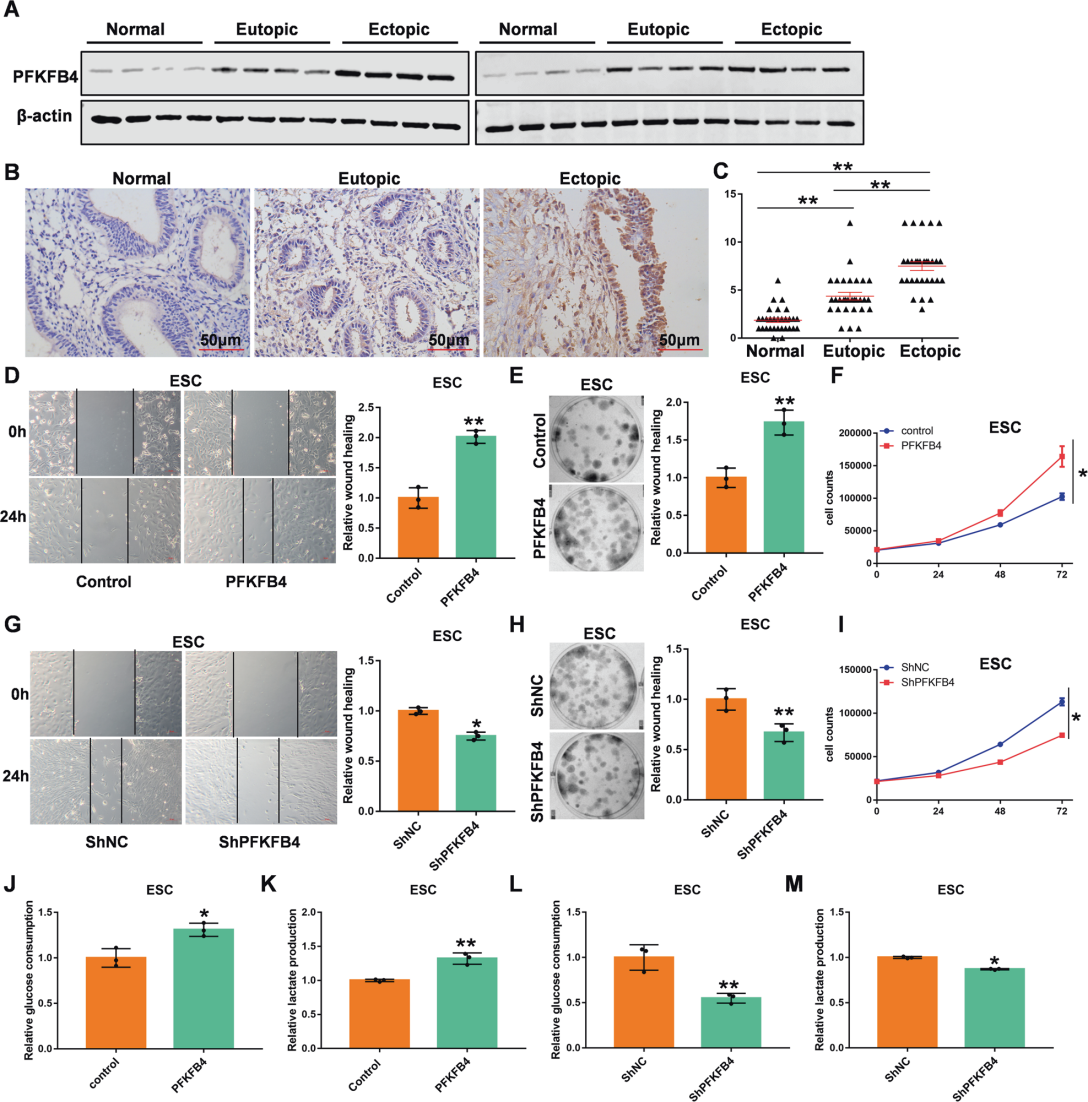
PFKFB4 is closely related to the progression of EM. **A** PFKFB4 protein expression in endometriosis tissues by Western Blot. **B** PFKFB4 protein expression in endometriosis tissues by immunohistochemistry. **C** Composite scores of PFKFB4 staining in endometriosis tissues by immunohistochemistry. **D** Wound healing assay after PFKFB4 was overexpressed in ESC cells. **E** Clone formation of PFKFB4 overexpressed in ESC cells. **F** Cell proliferation of PFKFB4 overexpressed in ESC cells. **G** Wound healing assay after PFKFB4 was knocked out by shPFKFB4 in ESC cells. **H** Clone formation of PFKFB4 lower expressed by shPFKFB4 in ESC cells. **I** Cell proliferation of PFKFB4 lower expressed by shPFKFB4 in ESC cells. **J** The glucose consumption when PFKFB4 is overexpressed in ESC cells. **K** The lactate production when PFKFB4 is overexpressed in ESC cells. **L** The glucose consumption when PFKFB4 was knocked out by shPFKFB4 in ESC cells. **M** The lactate production when PFKFB4 was knocked out by shPFKFB4 in ESC cells. All experiments were repeated at least 3 times. *p < 0.05, **p < 0.01.

Studies have found that the increase in glycolysis level is closely related to the progress of EM [35]. After screening the key genes of glycolysis, we found that PFKFB4 was upregulated in the EM compared with normal (Fig. 1A). The same results were obtained in the immunohistochemistry of EM samples (Fig. 1B, C). To further explore the effect of PFKFB4 on EM, PFKFB4 expression was transformed in ESC and 11Z endometriosis cells. We found that PFKFB4 upregulated significantly promoted the EM cell’s migration ability (Figs. 1D and S1A). The clone forming ability (Figs. 1E and S1B) and proliferation ability (Figs. 1F and S1C) of endometriosis cells also increased significantly when PFKFB4 was overexpressed. In contrast, the migration ability, clonogenic ability, and cell proliferation ability of endometriosis cells decreased significantly when we knocked out the expression of PFKFB4 in endometriosis cells (Figs. 1G–I and S2A–C). We also tested the glycolytic ability of endometriosis cells. Interestingly, glucose consumption (Figs. 1J and S3A) and Lactic acid production (Figs. 1K and S3B) were upregulated in endometriosis cells when PFKFB4 was overexpressed. Consistently, the glucose consumption (Figs. 1L and S3C) and Lactic acid production (Figs. 1M and S3D) were reduced in endometriosis cells when PFKFB4 was knockdown. These data show that PFKFB4 is closely related to the progression of EM.

### PIM2 is a new pioneering adjoin protein of PFKFB4

**Fig. 2:**
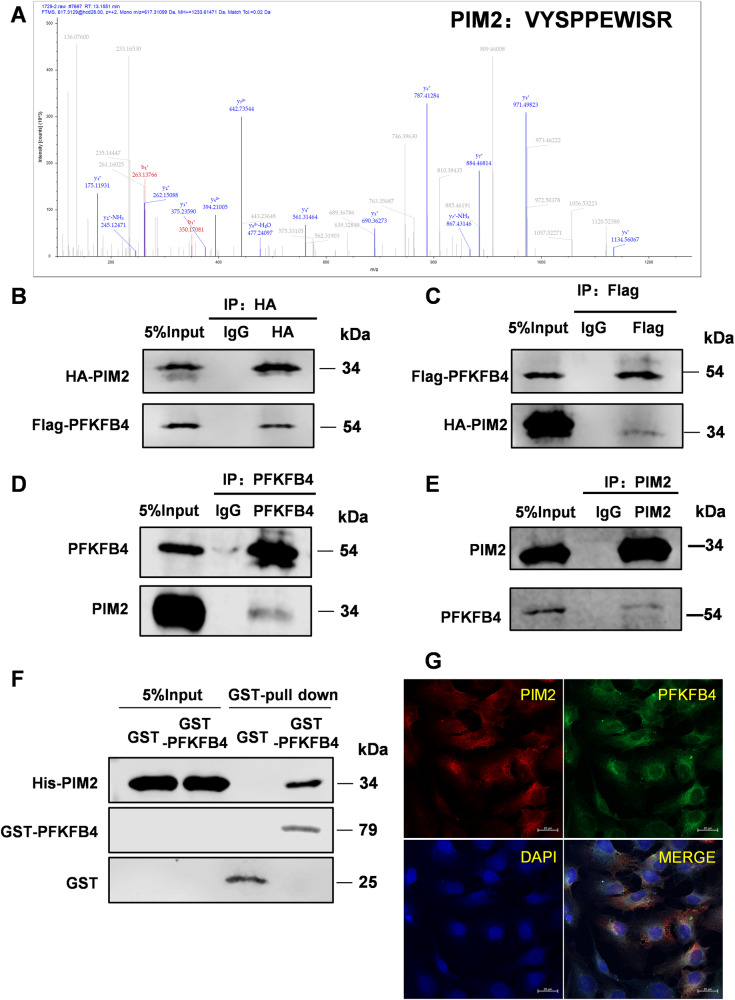
PIM2 is a novel binding partner of PFKFB4. **A** PIM2 may be a new binding protein of PFKFB4 by mass spectrometry analyses. **B**–**E** Co-immunoprecipitation was performed with indicated antibodies. HA-(**B**), Flag-(**C**), PFKFB4 (**D**), PIM2 (**E**). **F** GST-pull down was arranged to verify the in vitro binding of PFKFB4 to PIM2. **G** Confocal immunofluorescence microscopy was performed to analyze the localization of PIM2 and PFKFB4 in ESC cells. All experiments were repeated at least 3 times.

To explore the mechanisms central to what PFKFB4 functions in EM, we achieved mass spectrometry analyses of the immunoprecipitated PFKFB4 involved in EM cells and discovered that PIM2 may be a new pioneering adjoin protein of PFKFB4 (Fig. 2A and Supplementary excels 01). We validated the interaction between PFKFB4 and PIM2 in 293T cells and ESC cells by CO-IP assay (Fig. 2B–E). Moreover, the GST-pulldown experiment suggested that PIM2 could straight away interact with PFKFB4 (Fig. 2F). Then, we analyzed the expression regions of PIM2 and PFKFB4 in ESC cells by confocal analysis and found that they overlapped in the cytoplasm (Fig. 2G). These confirmed that PIM2 is a new pioneering adjoin protein of PFKFB4.

### PIM2 phosphorylates PFKFB4 at Thr140

**Fig. 3:**
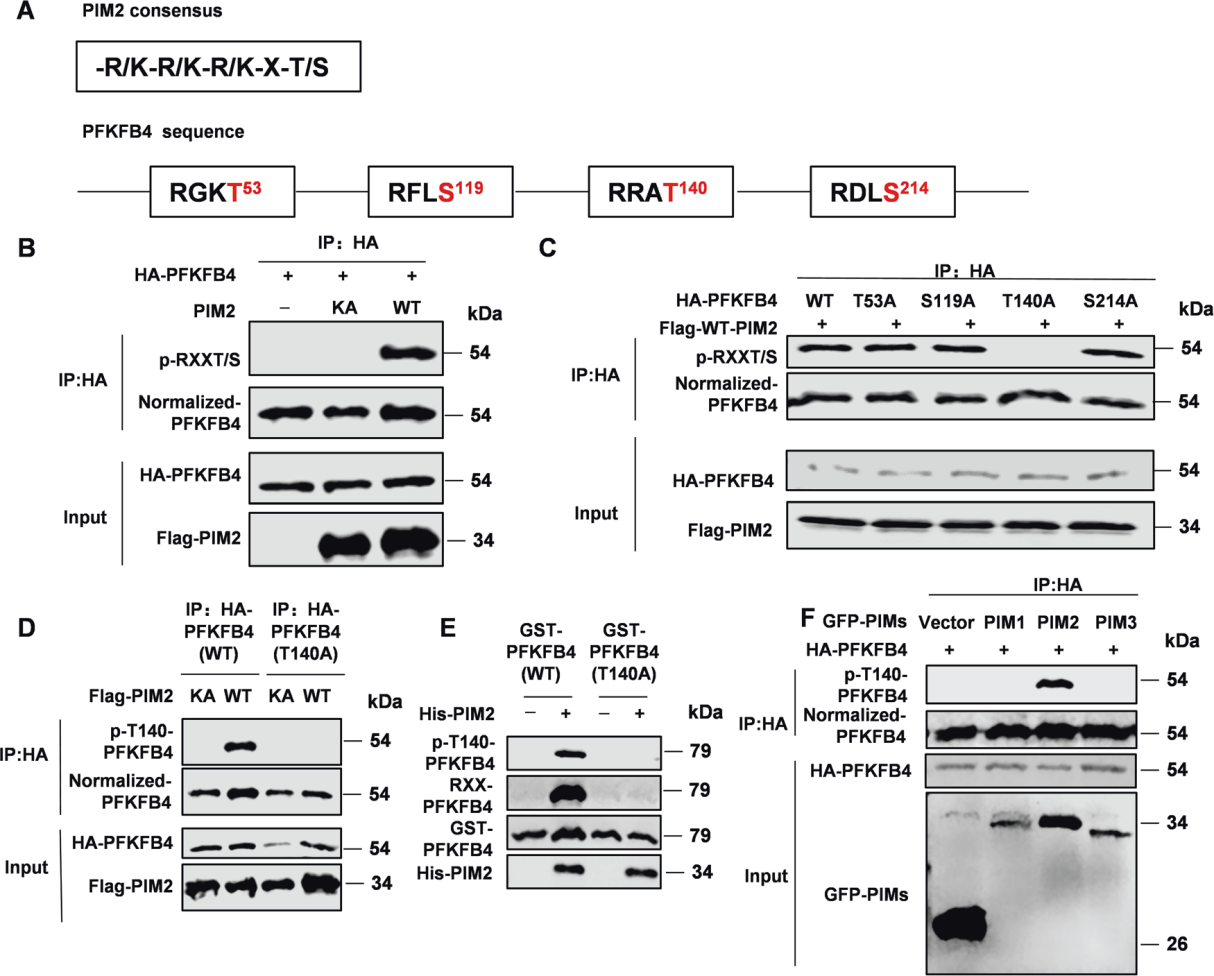
PIM2 phosphorylates PFKFB4 at Thr140. **A** PFKFB4 protein sequence predicted the binding site of PIM2. **B** HA-PFKFB4 and Flag-PIM2 (WT or KA) were overexpressed in HEK293T cells. Immunoprecipitation with an anti-HA antibody was performed. **C** HA-PFKFB4 (WT or mutant) and Flag-PIM2 (WT) were overexpressed in HEK293T cells. Immunoprecipitation with an anti-HA antibody was performed. **D** HA-PFKFB4 (WT or T140A) and Flag-PIM2 (WT or KA) were overexpressed in HEK293T cells. Immunoprecipitation with an anti-HA antibody was performed, followed by Western blot with indicated. **E** Purified GST-PFKFB4 was mixed with the indicated bacterially purified His-PIM2 proteins. An in vitro kinase assay was performed. **F** HA-PFKFB4 and GFP-PIMs (PIM1, PIM2, PIM3) were overexpressed in HEK293T cells. Total cell lysates were prepared. Immunoprecipitation with an anti-HA antibody was performed. All experiments were repeated at least 3 times.

We have previously demonstrated that PIM2 could recognize specific sequences of proteins to perform its kinase function [28, 31]. Then we analyzed the protein sequence of PFKFB4 and found 4 potential action sites (Fig. 3A). To further verify whether PIM2 can change the phosphorylation level of PFKFB4, we overexpressed both PIM2 and PFKFB4 in 293T cells, and found that wild-type PIM2 (WT-PIM2) can promote the phosphorylation of PFKFB4, while kinase-inactive PIM2 (KA-PIM2) cannot (Fig. 3B). In order to clarify specific action site, we made point mutation for the potential action site of PFKFB4. Repeated verification through relevant experiments found that site Thr140 was the action site of PIM2 (Fig. 3C). Next, we generated an antibody recognizing the PFKFB4 Thr140 phosphorylation specifically, and made a phosphorylation-inactive point mutation for Thr140(PFKFB4-T140A). As is shown in Fig. 3D, PIM2 could not act on PFKFB4-T140A. Consistently, in vitro experiments also confirmed that PIM2 could directly phosphorylate site Thr140 of PFKFB4 (Fig. 3E). In addition, the PIM group has three link members, PIM1, PIM2, and PIM3. Interestingly, only PIM2 was able to alter the phosphorylation of PFKFB4 (Fig. 3F). In summary, PIM2 could phosphorylate PFKFB4 at Thr140.

### PFKFB4 protein stability is regulated by PIM2

**Fig. 4:**
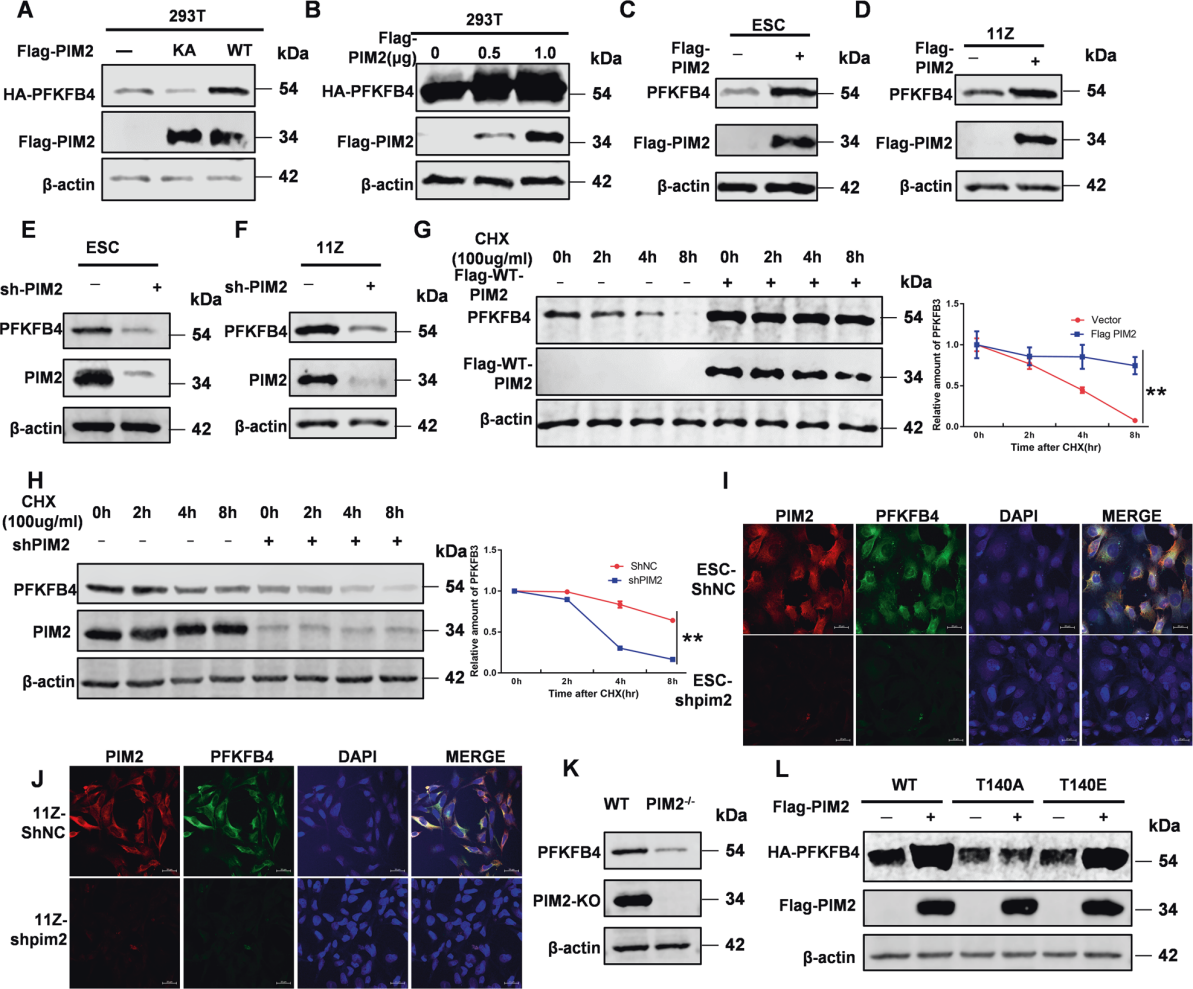
PFKFB4 protein stability is regulated by PIM2. **A** HA-PFKFB4 and Flag-PIM2 (WT or KA) were overexpressed in HEK293T cells for 72 h. Western blot analysis tested the expression of PIM2 and PFKFB4. **B** HA-PFKFB4 and Flag-PIM2 (0, 0.5, 1.0 μg) were overexpressed in HEK293T cells for 72 h. Western blot analysis tested the expression of PIM2 and PFKFB4. **C**, **D** Flag-PIM2 was overexpressed in ESC or 11Z cells for 72 h. Western blot analysis tested the expression of PIM2 and PFKFB4. **E**, **F** PIM2 was knocked out by shPFKFB4 in ESC or 11Z cells for 72 h. Western blot analysis tested the expression of PIM2 and PFKFB4. **G** HA-PFKFB4 and Flag-PIM2 plasmids were overexpressed in ESC cells and then treated with CHX for the indicated time. Total cell lysates were prepared. Western blot analysis tested the expression of PIM2 and PFKFB4. **H** PIM2 was knocked out by shPFKFB4 in ESC cells, and then treated with CHX for the indicated time. Total cell lysates were prepared. Western blot analysis tested the expression of PIM2 and PFKFB4. **I**, **J** PIM2 was knocked out by shPFKFB4 in ESC and 11Z cells. Confocal immunofluorescence microscopy was performed to analyze the localization of PIM2 and PFKFB4. **K** Detection of PIM2 and PFKFB4 expression in PIM2 knockout mice by Western Blot. **L** Flag-PIM2 and HA-PFKFB4 (WT, T140A, or T140E) were overexpressed in ESC cells. Western blot analysis tested the expression of PIM2 and PFKFB4. All experiments were repeated at least 3 times. **p < 0.01.

In order to explore the regulation of PIM2 on the stability of PFKFB4 protein, we overexpressed PIM2 and PFKFB4 in 293T cells. The results were shown in Fig. 4A, Flag-WT-PIM2 promoted the PFKFB4 expression compared with KA-PIM2. Interestingly, the expression of PFKFB4 was closely related to the dose of PIM2 (Fig. 4B). Then we verified it in endometriosis cells. When the expression of PIM2 increased, the expression of PFKFB4 also increased (Fig. 4C, D). Simultaneously, PFKFB4 expression was decreased when PIM2 expression was knocked down by shPIM2 in endometriosis cells (Fig. 4E, F). To further clarify the mechanism of PIM2 which regulated the stability of PFKFB4, the protein synthesis inhibitor cycloheximide was used in ESC cells for various periods of time. As was shown in Fig. 4G, PIM2 could delay the degradation of PFKFB4 through a CMA pathway. When the expression of PIM2 was knocked down by shPIM2, the degradation rate of PFKFB4 increased significantly (Fig. 4H). Then, we analyzed the change regions between PIM2 and PFKFB4 in endometriosis cells by confocal analysis and found that PFKFB4 protein level expression was decreased when shPIM2 was used to knock down PIM2 expression (Fig. 4I, J). PIM2 knockout mice were constructed in our laboratory [18]. We tested the embryonic fibroblasts of PIM2−/− mice and found that the protein expression of PFKFB4 also decreased significantly (Fig. 4K). To verify whether the Thr140 site works on protein stability, PFKFB4 Thr140 was mutated to phosphorylation inactivation (T140A) or continuous phosphorylation (T140E). As shown in Fig. 4L, PFKFB4 T140A protein stability was not changed significantly while PFKFB4 WT and T140E protein levels enhanced when PIM2 existed. Above all, we concluded that PFKFB4 protein stability was regulated on Thr140 via the CMA pathway by PIM2.

### PFKFB4 Thr140 phosphorylation promotes cell proliferation, clone formation, and cell migration

**Fig. 5:**
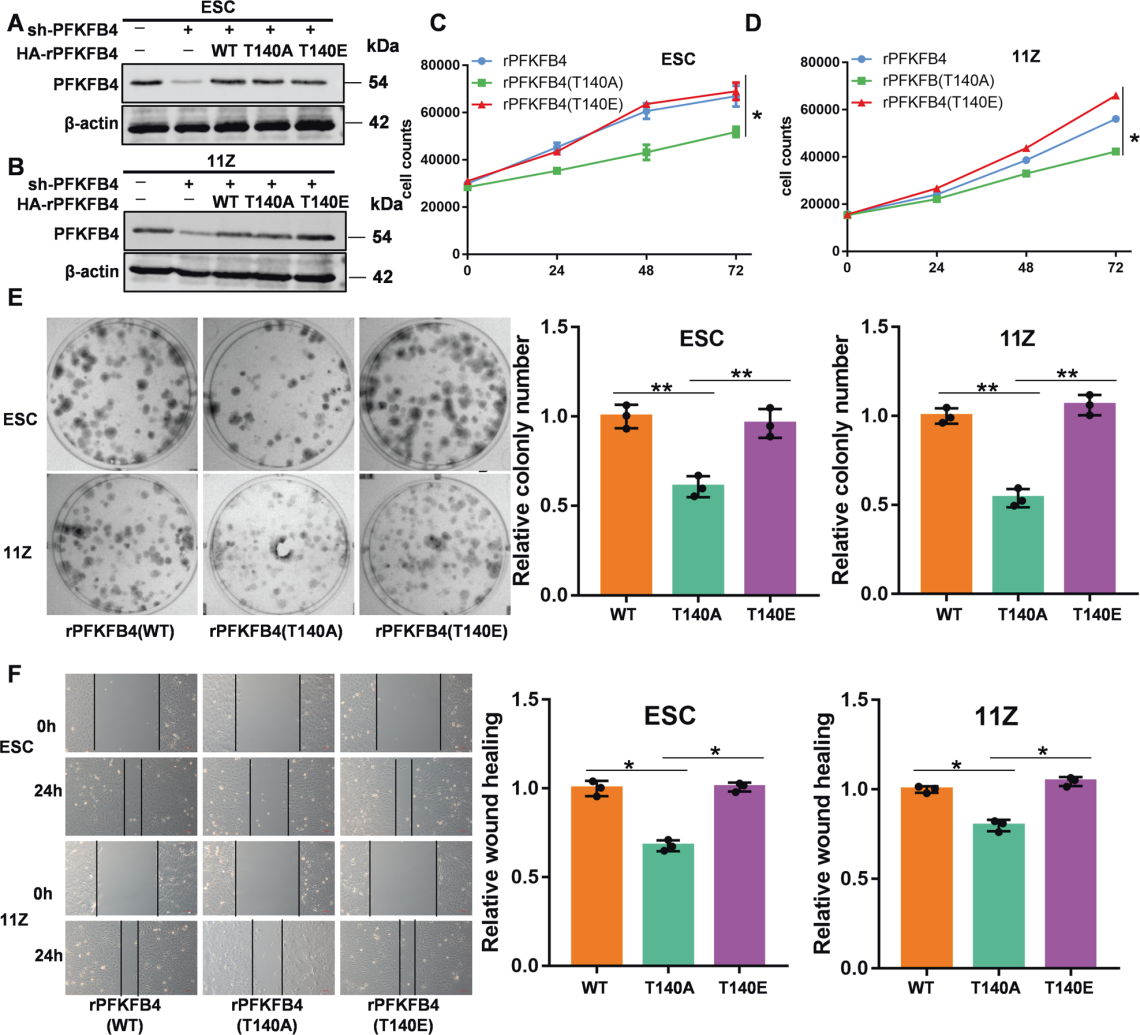
PFKFB4 Thr140 phosphorylation promotes the biological behavior of EM. **A**, **B** PFKFB4 was knocked out by shPFKFB4, and then re-expressed in endometriosis cells. **C**, **D** Cell proliferation of PFKFB4 rHAPFKFB4 (WT, T140A, or T140E) endometriosis cells. **E** Clone formation of PFKFB4 rHAPFKFB4 (WT, T140A, or T140E) endometriosis cells. **F** Wound healing assay of PFKFB4 rHAPFKFB4 (WT, T140A, or T140E) endometriosis cells. All experiments were repeated at least 3 times. *p < 0.05, **p < 0.01.

To verify the effect of the PFKFB4 Thr140 site on endometriosis cells, we knocked out endogenous PFKFB4 expression and re-expressed them in endometriosis cells above (Fig. 5A, B). The cell proliferation ability of rPFKFB4(T140A) was significantly lower than rPFKFB4 (WT or T140E) which means PFKFB4 Thr140 phosphorylation promoted cell proliferation (Fig. 5C, D). Moreover, the clone forming ability (Fig. 5E) and migration ability (Fig. 5F) of PFKFB4 Thr140 phosphorylation induced by PIM2 were also increased. So PFKFB4 Thr140 phosphorylation was closely related to the biological behavior of EM.

### PIM2 and PFKFB4 Thr140 phosphorylation promote glycolysis in endometriosis

**Fig. 6:**
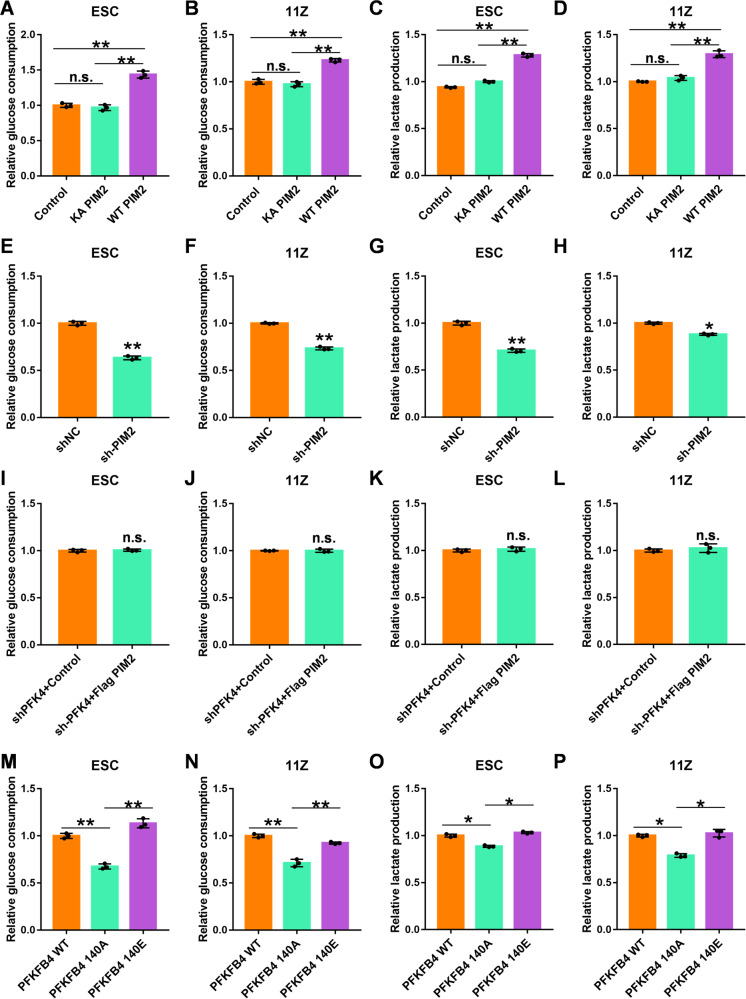
PIM2 and PFKFB4 Thr140 phosphorylation promote glycolysis in endometriosis. **A–D** The glucose consumption and the lactate production when PIM2 (KA and WT) overexpressed in endometriosis cells. **E–H** The glucose consumption and lactate production when PIM2 was knocked out by shPIM2 in endometriosis cells. **I–L** The glucose consumption and the lactate production when PFKFB4 was knocked out by shPFKFB4 and then overexpressed PIM2 protein levels. **M–P** The glucose consumption and the lactate production PFKFB4 were knocked out by shPFKFB4 and then re-expressed in endometriosis cells. All experiments were repeated at least 3 times. *p < 0.05, **p < 0.01. ns was not significant.

We have previously demonstrated that PIM2 promoted glycolysis in breast cancer [31], however, it is unclear whether it can play a role in EM. Here we tested the glucose consumption and lactate production after changing PIM2 expression in endometriosis cells. The results were shown in Fig. 6A–D, the glucose consumption and lactate production were increased in ESC and 11Z cells when PIM2 was overexpressed. The glycolysis decreased significantly when PIM2 was knocked down by shPIM2 (Fig. 6E–H). Interestingly, when we knocked down PFKFB4 in endometriosis cells by shPFKFB4 and then overexpressed PIM2 protein levels, the glucose consumption, and lactate production were not clearly changed (Fig. 6I–L). This indicates that the glycolytic regulation capacity of PIM2 was dependent on PFKFB4 in EM. Because PIM2 could alter the phosphorylation of PFKFB4 Thr140, we tested the glycolytic capacity of PFKFB4 with altered phosphorylation at Thr140. Surprisingly, phosphorylation at Thr140 increased glucose consumption and lactate production in endometriosis cells while the T140A PFKFB4 mutant could not function (Fig. 6M–P). Therefore, PIM2 and PFKFB4 Thr140 phosphorylation promotes glycolysis in endometriosis.

### PIM2 is truly relative with PFKFB4 in endometriosis in vivo

**Fig. 7:**
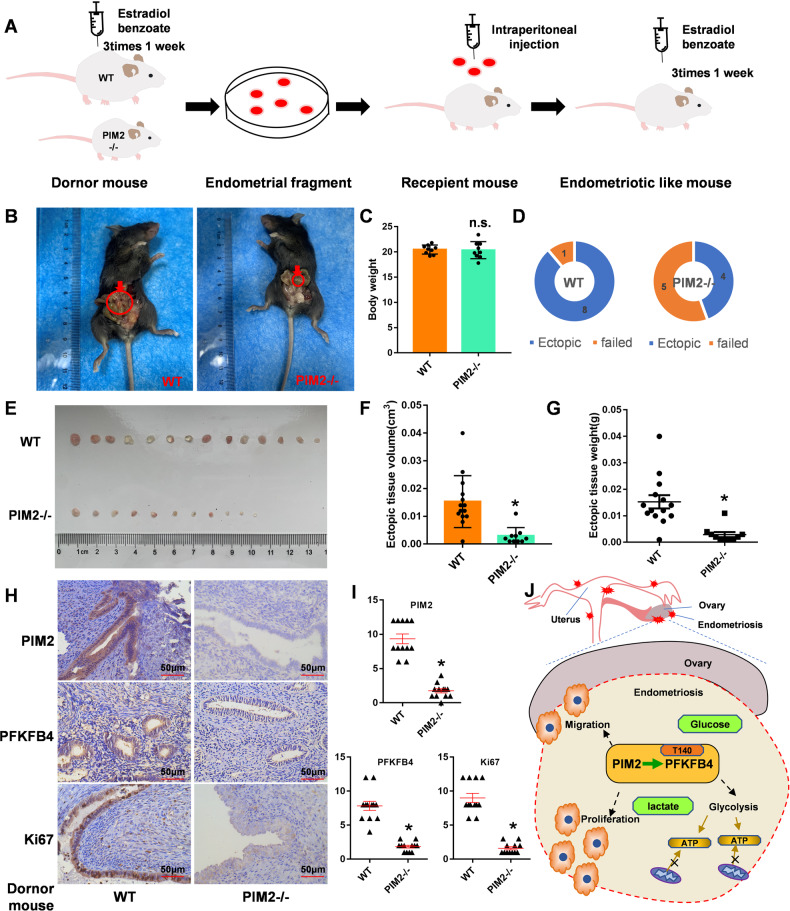
PIM2 expression is positively correlated with PFKFB4 in endometriosis in vivo. **A** Schematic diagram of a mouse model of endometriosis using PIM2-knockout endometrial tissue as a donor. **B** The two-mouse model of endometriosis. **C** The body weight of these two mice model groups. **D** The success rate of ectopic foci of two-mouse endometriosis model. **E** The endometriotic tissues of two-mouse endometriosis model. **F** The volume of the two-mouse endometriosis model. **G** The weight of a two-mouse model of endometriosis. **H** Immunohistochemical expression of PIM2, PFKFB4, and Ki67 in two-mouse endometriosis model. **I** Composite scores of PIM2, PFKFB4, and Ki67 in a two-mouse endometriosis model. **J** A working model that schematic diagram of PIM2 regulating EM glycolysis and paclitaxel resistance through PFKFB4 Thr140 site. *p < 0.05, ns was not significant.

To further determine that PIM2 can regulate PFKFB4 in vivo, we utilized PIM2-knockout endometrial tissue as a donor to construct a mouse model of endometriosis (Fig. 7A). Both the two-mouse model of endometriosis were shown in Fig. 7B. Although the body weight of these two mice model groups did not change significantly (Fig. 7C), the success rate of ectopic foci in the PIM−/− sort out was essentially less than a lapse in the normal group (Fig. 7D). We also found significantly smaller endometriotic tissue in PIM2−/− mouse model compared with the normal group (Fig. 7E). Similarly, the volume and weight of PIM2−/− group were also significantly reduced (Fig. 7F, G). We performed HE staining on the obtained ectopic foci to confirm that they were endometrial tissues (Fig. S4A). Then we detected the expression of PIM2, PFKFB4, and Ki67 in ectopic foci of two groups of mouse models by immunohistochemical experiments. As was shown in Fig. 7H, I, the expression of PIM2, PFKFB4, and Ki67 were lower expressions in PIM2−/− group. Finally, we detected the expression of PIM2 and PFKFB4 in EM samples. The result shown in Fig. S4B, PFKFB4, and PIM2 were strongly expressed in Ectopic samples. Consistently, both two expressions were positively correlated (Fig. S4C). The findings show that PIM2 expression was positively correlated with PFKFB4 in endometriosis in vivo.

## Discussion

The increase in glycolysis level is closely related to the progress of EM [35, 36]. Peritoneal mesothelial cells were isolated away from the pelvic peritoneum of women with EM and found the cells exhibited significantly lower mitochondrial respiration and higher levels of glycolysis [37]. The Lactate concentrations were as well in the long run selected in the peritoneal fluid of patients with EM [38]. At the same time, the related proteomic analysis found that ectopic endometrial stromal cells have extensive metabolic reprogramming and cancer-like changes, which have striking similarities with tumorigenesis [35, 39, 40]. Interestingly, the glycolysis inhibitor reduces the mouse peritoneal fluid lactate concentration and the size of endometriotic lesions in the mouse model of EM [37]. These findings provide a basis for non-contraceptive therapy in metabolism. In view of the importance of glycolysis in EM, related glycolysis research and non-contraceptive therapy targeting glycolysis have been hot in recent years [36, 41, 42]. Here, we made a study on PFKFB4 which was one of the key regulators of glycolysis to explore the potential pathogenesis of EM and provide a theoretical basis for better diagnosis and treatment of EM.

PFKFB4 belongs to the PFK2 family. PFKFB4 is a bifunctional enzyme that can play both kinase and phosphatase activities [12, 43]. PFKFB4 can perform both glycolytic and non-glycolytic regulatory functions [15, 44]. PFKFB4 is parts bustling in many basic processes, such as autophagy and transcriptional regulation in a glycolysis-independent manner [45, 46]. The lofty phosphatase function of PFKFB4 depths safeguards ailment cells from excessive oxidative stress. Knocking down the expression of PFKFB4 makes reactive oxygen species easy to accumulate in cancer cells and further induces autophagy [45]. PFKFB4 can also be connected with the SRC- 3 and provoke intended genes of SRC-3-driven participation in non-oxidative PPP and purine anabolism [11]. In our study, we explored the glycolytic regulatory function of PFKFB4 in EM. We performed a new phosphorylation site Thr140 of PFKFB4 which played a role in regulatory significance for glycolysis and biological behavior in EM. PFKFB4 may be a potential target for EM diagnosis and therapy.

There are many ways that PIM2 participates in the regulation of biological processes, and the regulation of glycolysis is one of the most important mechanisms [22]. PIM kinases can promote cellular hyper glycolysis by mediating the phosphorylation of K-Ras, further changing the lactate environment in local tissues, regulating cell survival, and blocking immune cell function [47]. Our previous research has found that PIM2 can promote glycolysis by phosphorylating Thr473-HK2 and Ser478-PFKFB3 [28, 31]. PIM2 can also act on the glycolysis key enzyme PKM2 to promote the pentose phosphate pathway and thus affect the level of glycolysis in cells [48, 49]. Here, we illustrated a new mechanism that PIM2 simulated a central upstream partnership in the regulation of PFKFB4, and reveal a novel means of PIM2-PFKFB4 setting EM growth.

Our findings demonstrate that abnormally high expression of PFKFB4 was closely related to EM’s progression. Identification by mass spectrometry revealed that PIM2 may be a potential binding protein of PFKFB4. Then PIM2 phosphorylated site Thr140 of PFKFB4 was confirmed by biochemical methods. PIM2 also could enhance PFKFB4 protein expression through the ubiquitin-proteasome pathway. Moreover, PIM2 expression is positively correlated with PFKFB4 in endometriosis in vivo. What’s more, phosphorylation of PFKFB4 on Thr140 promoted EM glycolysis and cell growth. Above all, phosphorylation of PFKFB4 resulted in EM glycolysis and development via PIM2 (Fig. 7J). Endometriosis exists in a specific hypoxic microenvironment [50]. Endometrial cells can survive in hypoxic environments by upgrading their metabolic properties [51]. The metabolic transition between aerobic glycolysis and oxidative phosphorylation plays a major role in the occurrence and development of endometriosis, and the change of its signal pathway can become a feasible target for therapeutic intervention. Interestingly, our research was aimed at the glycolysis of endometriosis [52]. Our research provided new theoretical support for further clarifying the reprogramming of EM glucose metabolism, and provided new clues for exploring non-contraceptive treatments for EM.

### Supplementary information

Supplementary Data Figure Legends

Supplementary original western blots

aj-checklist-CDDIS-22-1287

suppllent excels 01

Supplementary Figure S1

Supplementary Figure S2

Supplementary Figure S3

Supplementary Figure S4

## Data Availability

The authors declared that all the data and materials are available on reasonable request.
