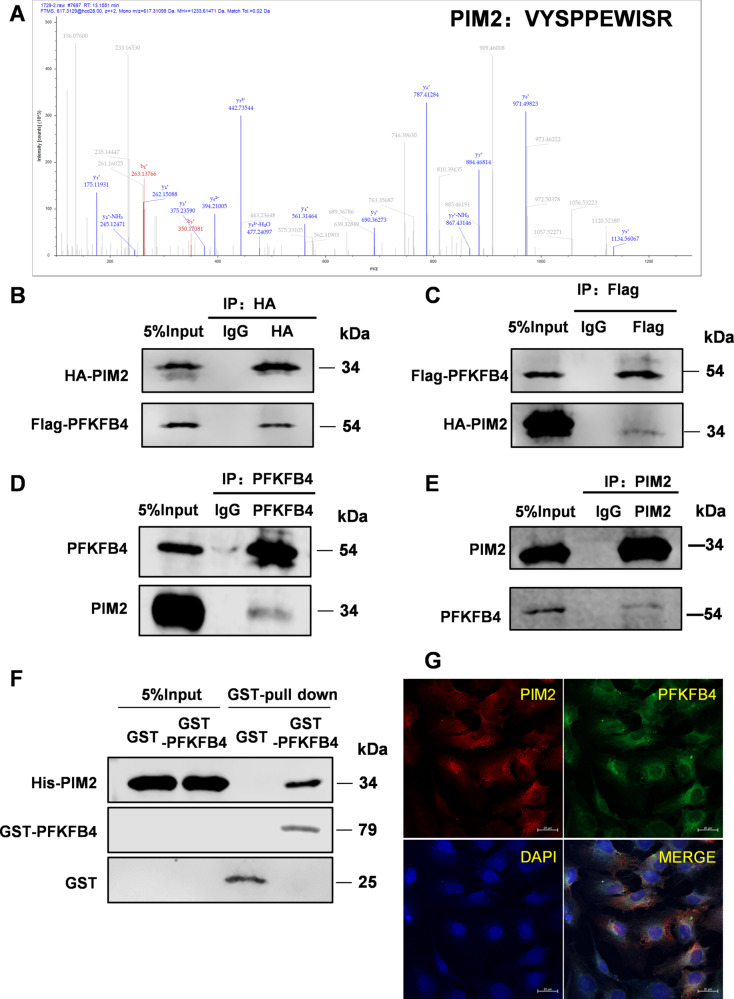# Correction: Phosphorylation of PFKFB4 by PIM2 promotes anaerobic glycolysis and cell proliferation in endometriosis

**DOI:** 10.1038/s41419-024-06613-w

**Published:** 2024-07-02

**Authors:** Chao Lu, Pengyun Qiao, Ruihai Fu, Yadi Wang, Jiayi Lu, Xi Ling, Lu Liu, Yujun Sun, Chune Ren, Zhenhai Yu

**Affiliations:** Department of Reproductive Medicine, Affiliated Hospital of Weifang Medical University, Weifang, Shandong Province P. R. China

**Keywords:** Phosphorylation, Phosphorylation

Correction to: *Cell Death & Disease* 10.1038/s41419-022-05241-6, published online 15 September 2022.

During checking the published paper again, the authors found that the original version of this article contained a mistake in Figure 2E. The correct image has now been incorporated and are given below.

This correction does not change the description, interpretation, or the original conclusions of the manuscript. The authors apologize for the mistakes and any inconvenience caused.

The original article has been corrected.