# Supplementary material for: Phosphorylation of PFKFB4 by PIM2 promotes anaerobic glycolysis and cell proliferation in endometriosis
Source: Cell Death Dis. 2022 Sep 15;13(9):790. doi: 10.1038/s41419-022-05241-6 (PMC9477845; doi:10.1038/s41419-022-05241-6)
Supplement: Supplementary file 1 — Supplementary Data Figure Legends [file 41419_2022_5241_MOESM1_ESM.doc]

**List of Supplementary Data Figure Legends**

**Supplement Fig. S1** **PFKFB4 is closely related to the progression of EM**

**A** Wound healing assay after PFKFB4 was overexpressed in 11Z cells. **B** Clone formation of PFKFB4 overexpressed in 11Z cells. **C** Cell proliferation of PFKFB4 overexpressed in 11Z cells.

All experiments were repeated at least 3 times. *p<0.05, **p<0.01.

**Supplement Fig. S2 PFKFB4 is closely related** to **the progression of EM**

**A** Wound healing assay after PFKFB4 was knocked out by shPFKFB4 in 11Z cells. **B** Clone formation of PFKFB4 lower expressed by shPFKFB4 in 11Z cells. **C** Cell proliferation of PFKFB4 lower expressed by shPFKFB4 in 11Z cells.

All experiments were repeated at least 3 times. *p<0.05, **p<0.01.

**Supplement Fig. S3 The abnormal expression of PFKFB4 on glycolysis in endometriosis**

**A** The glucose consumption when PFKFB4 overexpressed in 11Z cells. **B** The lactate production when PFKFB4 overexpressed in 11Z cells. **C** The glucose consumption when PFKFB4 was knocked out by shPFKFB4 in 11Z cells. **D** The lactate production when PFKFB4 was knocked out by shPFKFB4 in 11Z cells.

All experiments were repeated at least 3 times. **p<0.01.

**Supplement Fig. S4 PIM2 expression is positively correlated with PFKFB4 in endometriosis in *vivo***

**A** HE staining on the obtained ectopic foci. **B** PFKFB4 and PIM2 protein expression in endometriosis tissues by Immunohistochemistry. **C** Pearson correlation analysis of PFKFB4 and PIM2 semi-quantitative staining score.
