# Supplementary material for: Phosphorylation of PFKFB4 by PIM2 promotes anaerobic glycolysis and cell proliferation in endometriosis
Source: Cell Death Dis. 2022 Sep 15;13(9):790. doi: 10.1038/s41419-022-05241-6 (PMC9477845; doi:10.1038/s41419-022-05241-6)
Supplement: Supplementary file 2 — Supplementary original western blots [file 41419_2022_5241_MOESM2_ESM.docx]

**Supplementary original western blots**

**Figure 1 original western blots**

**
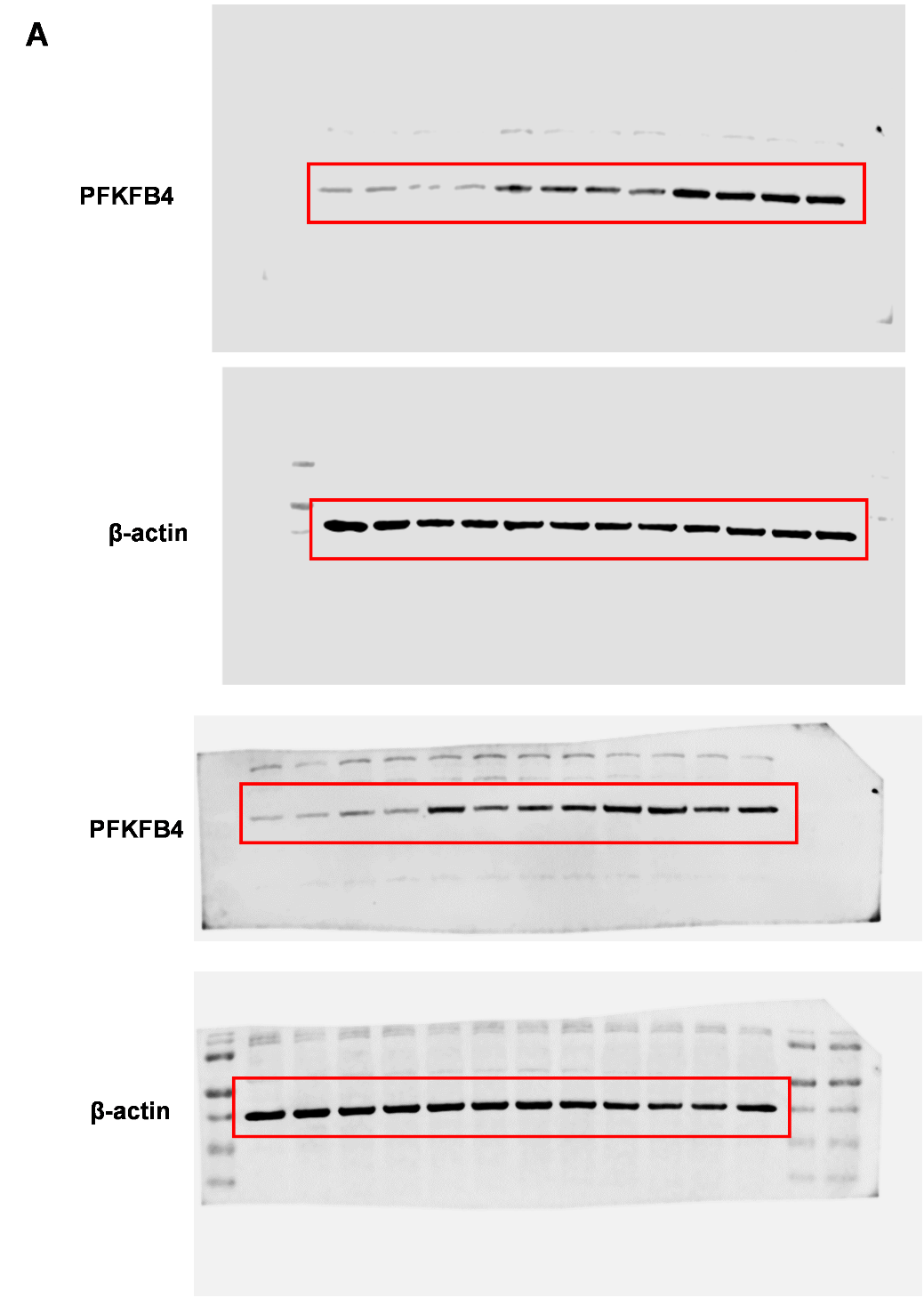
**

**Figure 2 original western blots**

**
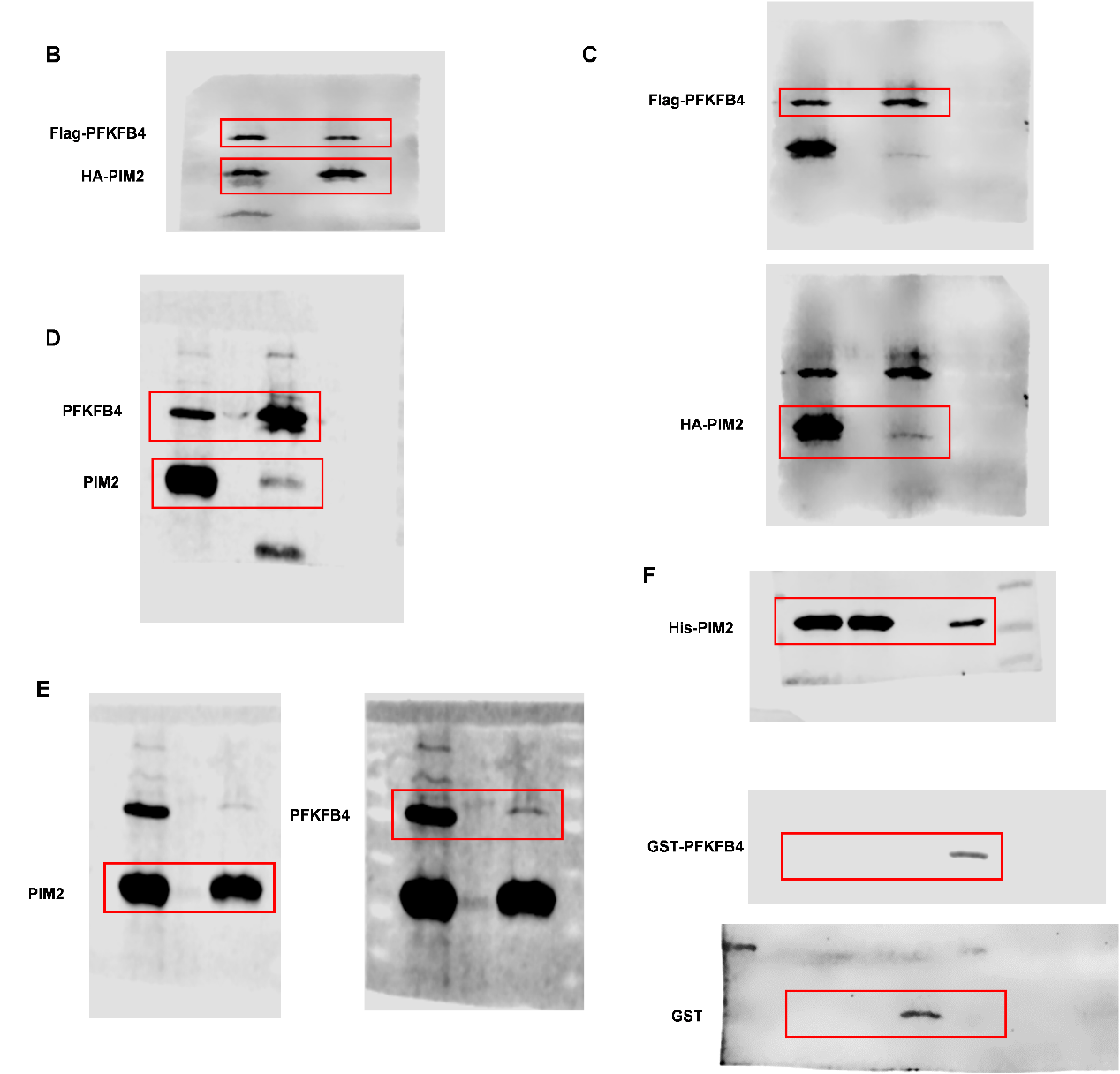
**

**Figure 3.1 original western blots**


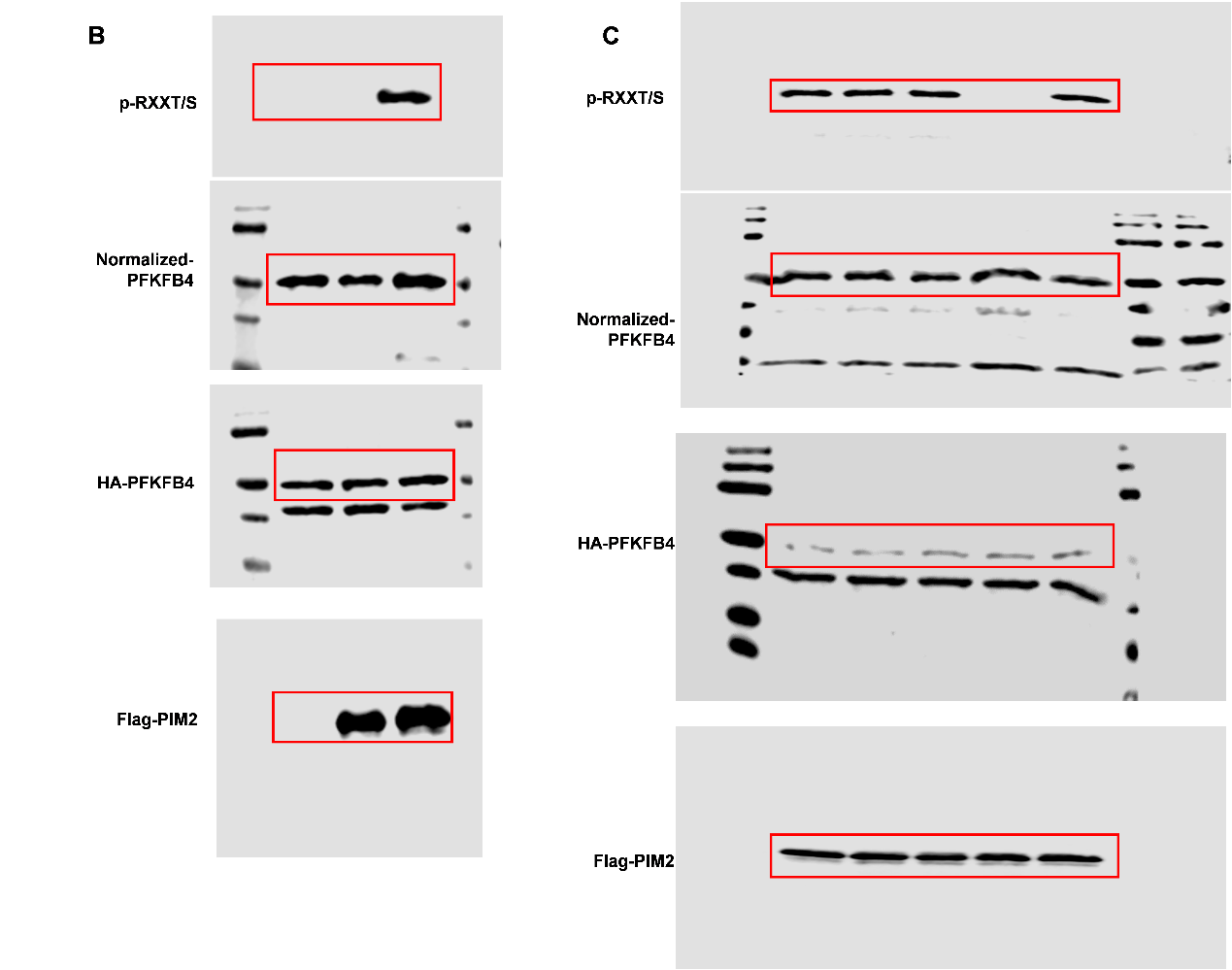


**Figure 3.2 original western blots**

**
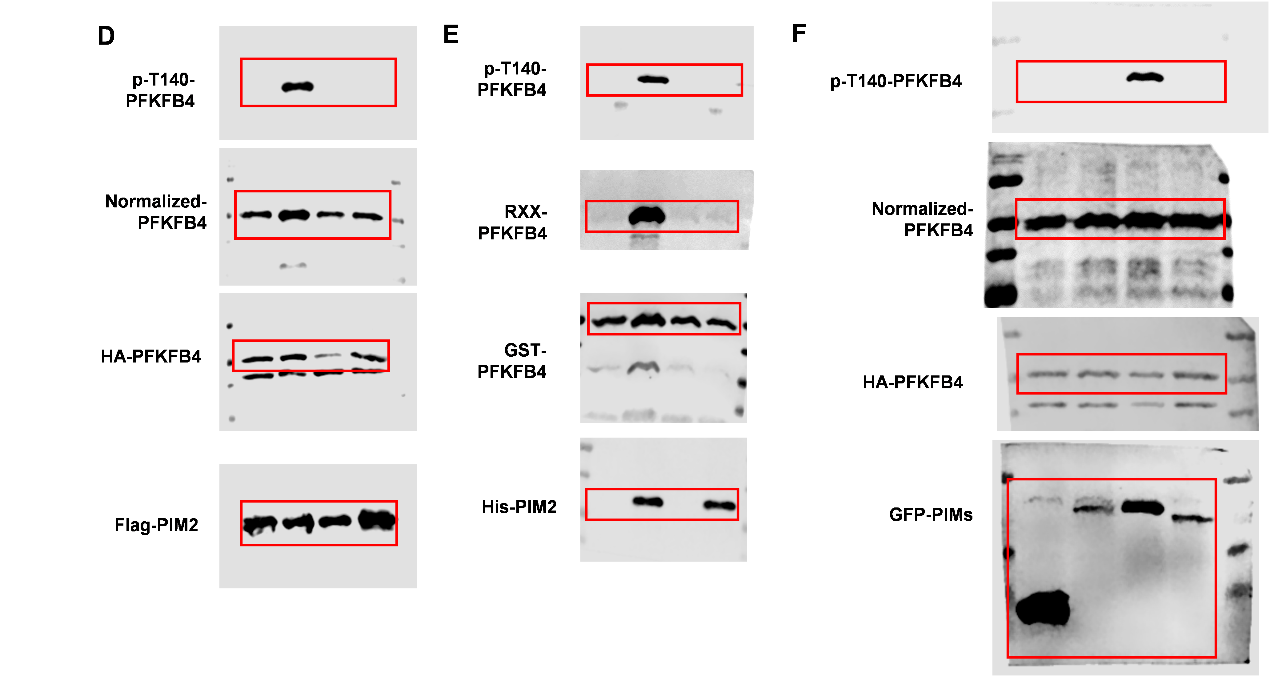
**

**Figure 4.1 original western blots**

**
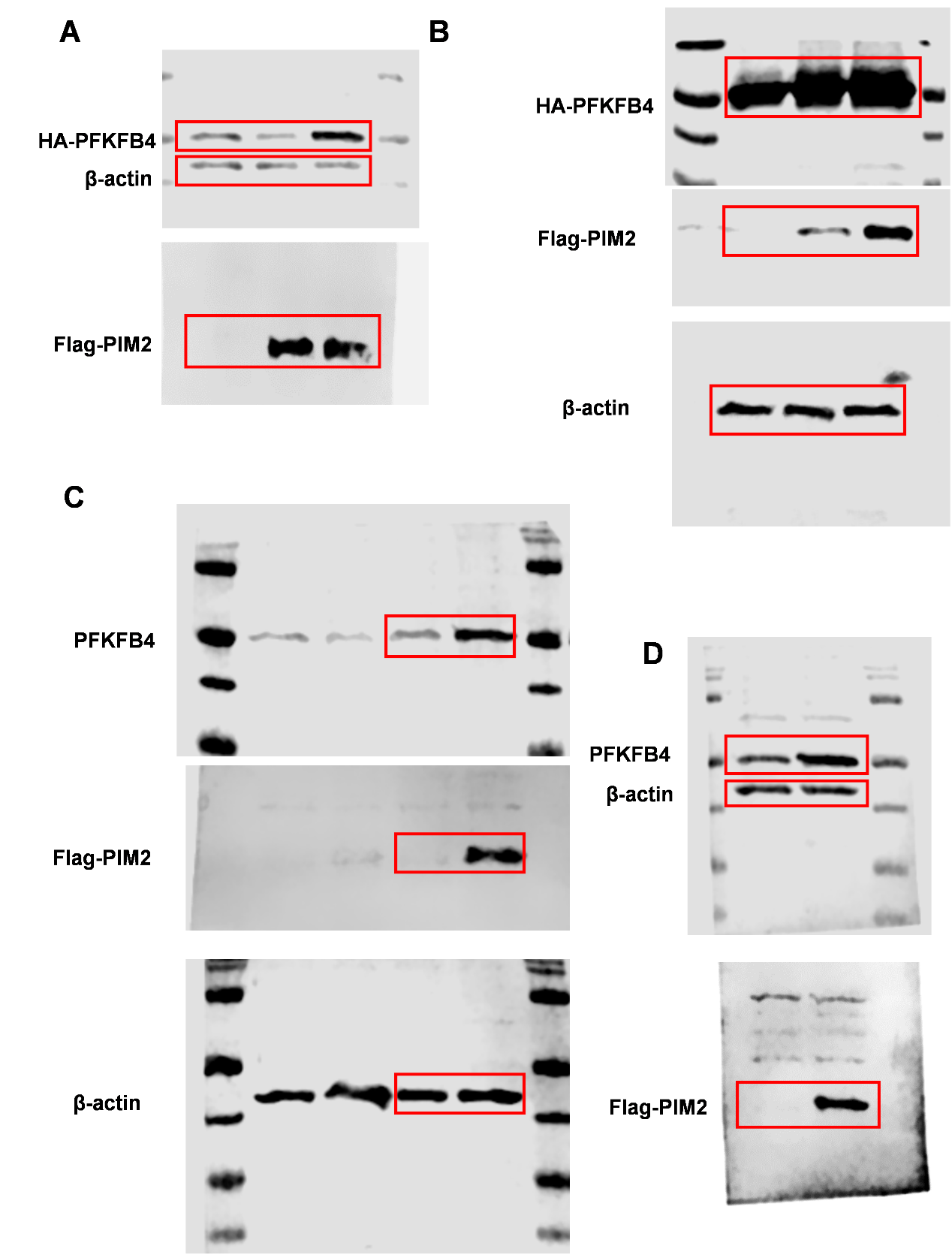
**

**Figure 4.2 original western blots**

**
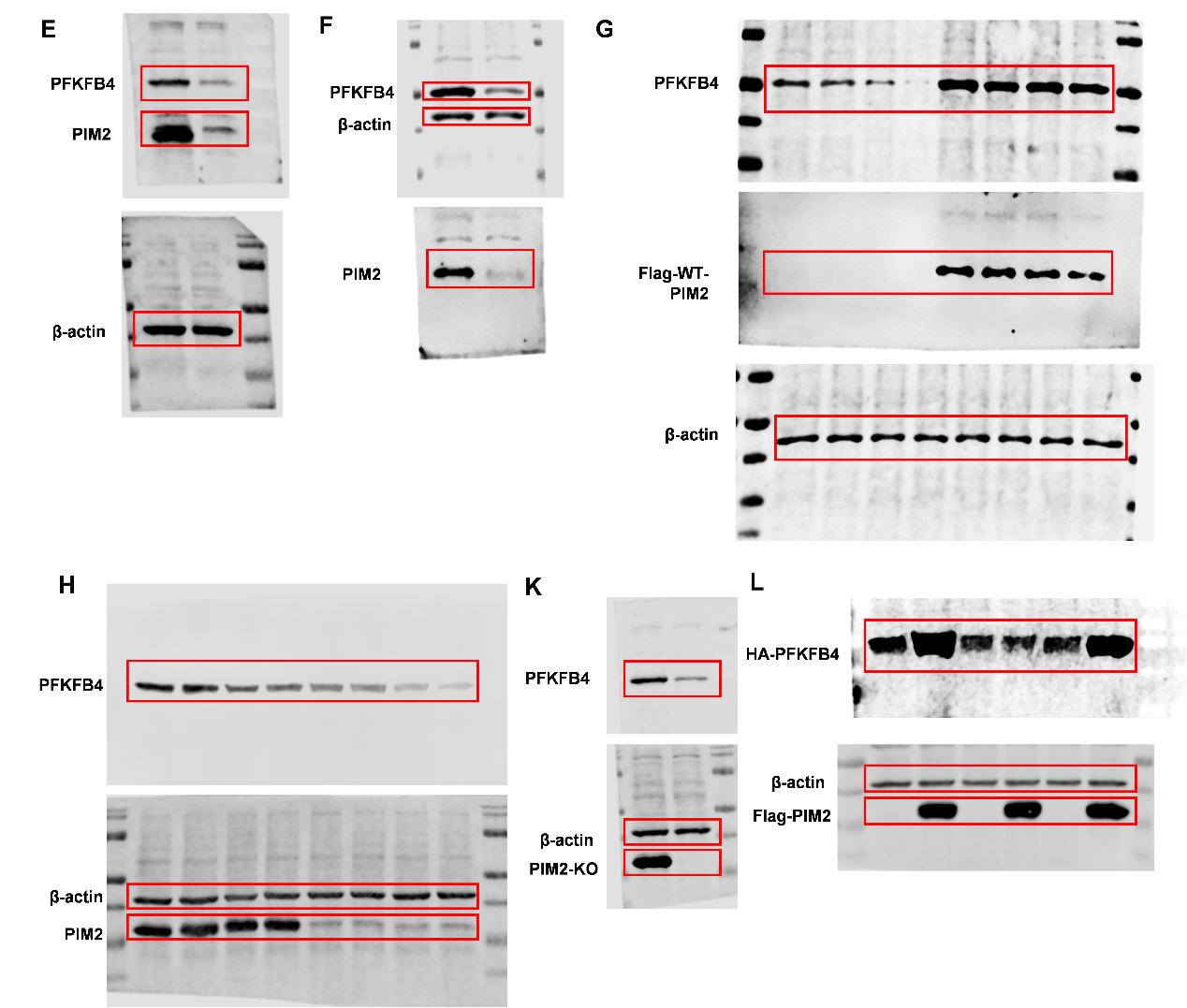
**

**Figure 5 original western blots**

**
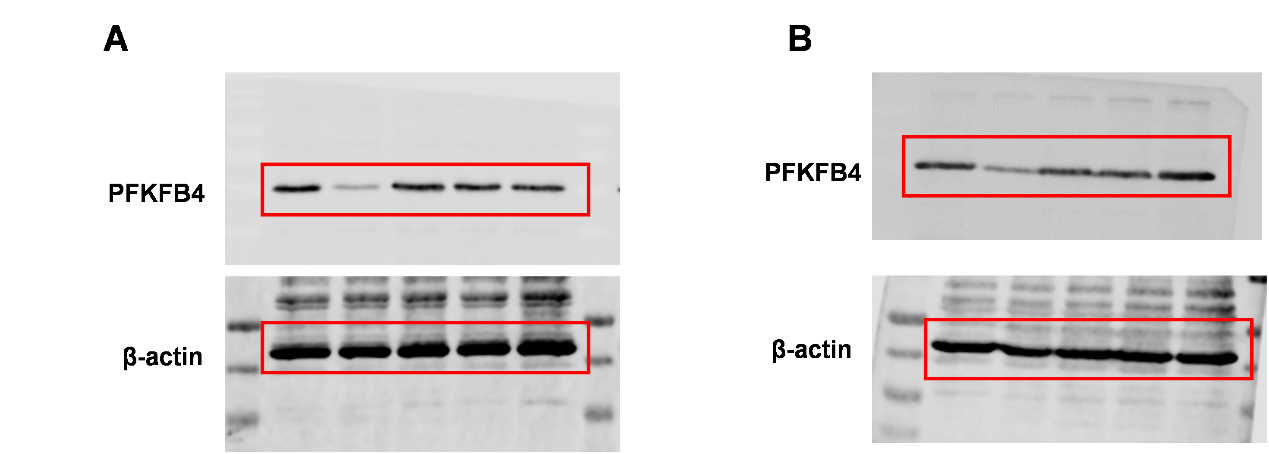
**
