# Supplementary figures and images for: Phosphorylation of PFKFB4 by PIM2 promotes anaerobic glycolysis and cell proliferation in endometriosis
Source: Cell Death Dis. 2022 Sep 15;13(9):790. doi: 10.1038/s41419-022-05241-6 (PMC9477845; doi:10.1038/s41419-022-05241-6)

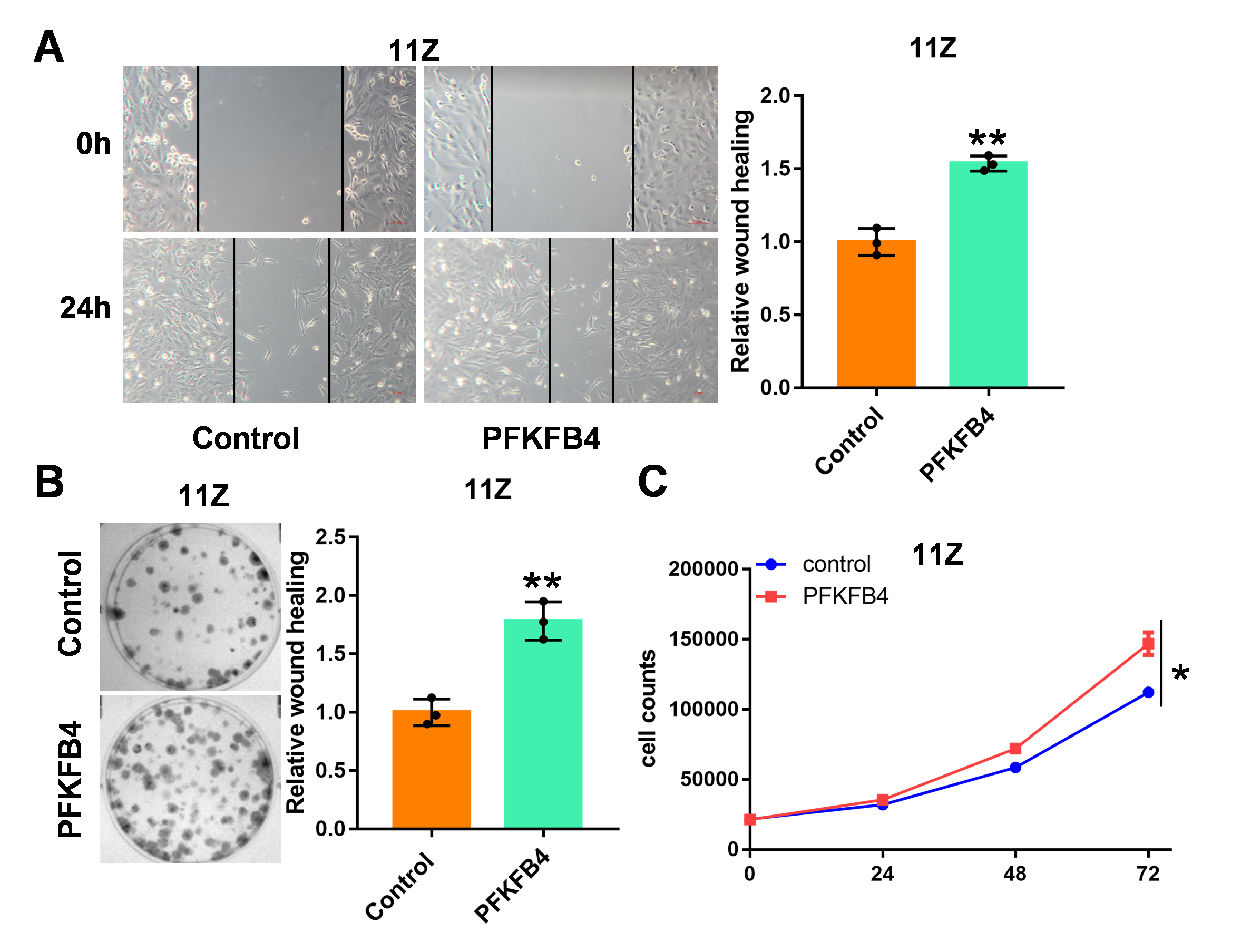

Supplement: Supplementary file 5 — Supplementary Figure S1 [file 41419_2022_5241_MOESM5_ESM.tif]

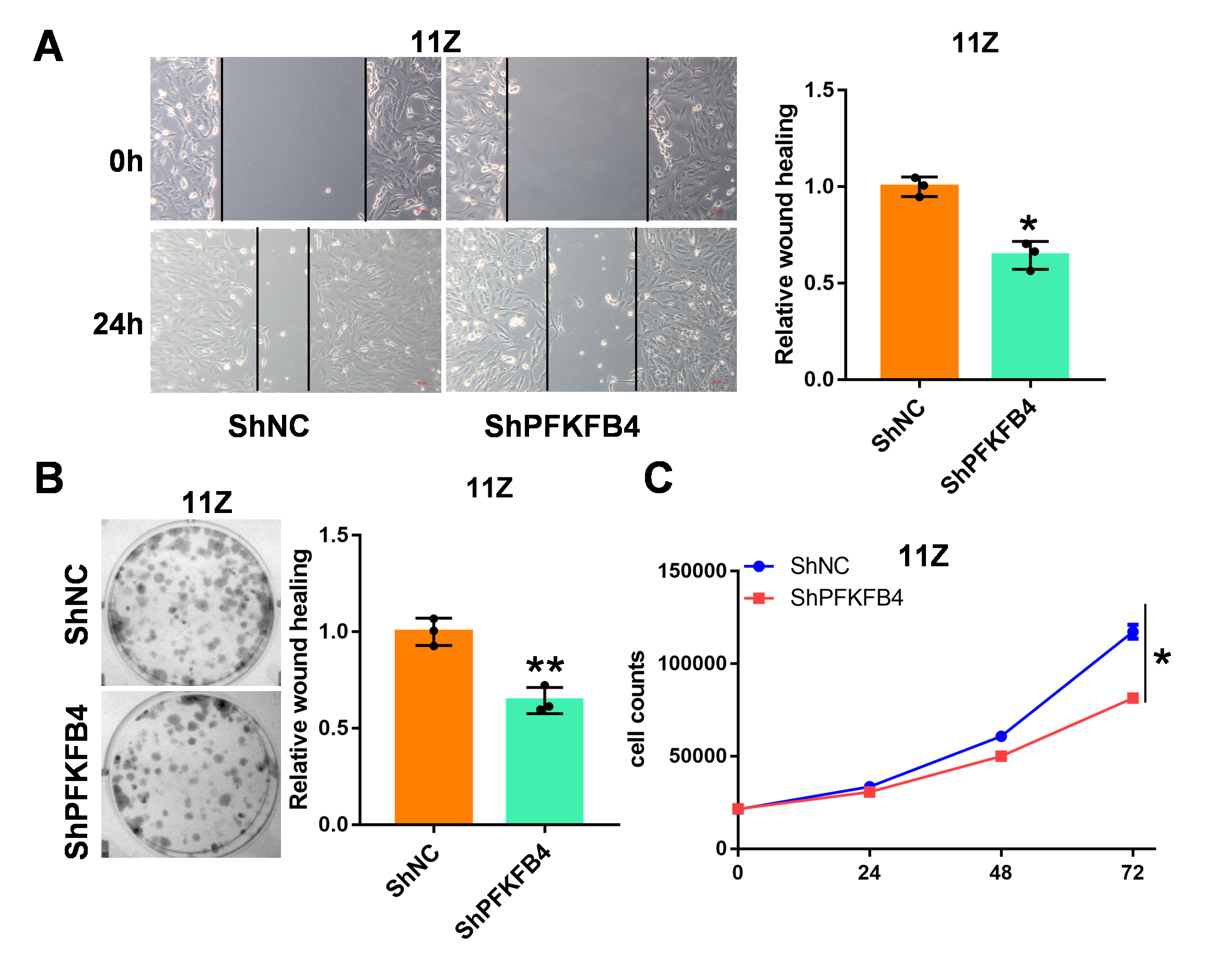

Supplement: Supplementary file 6 — Supplementary Figure S2 [file 41419_2022_5241_MOESM6_ESM.tif]

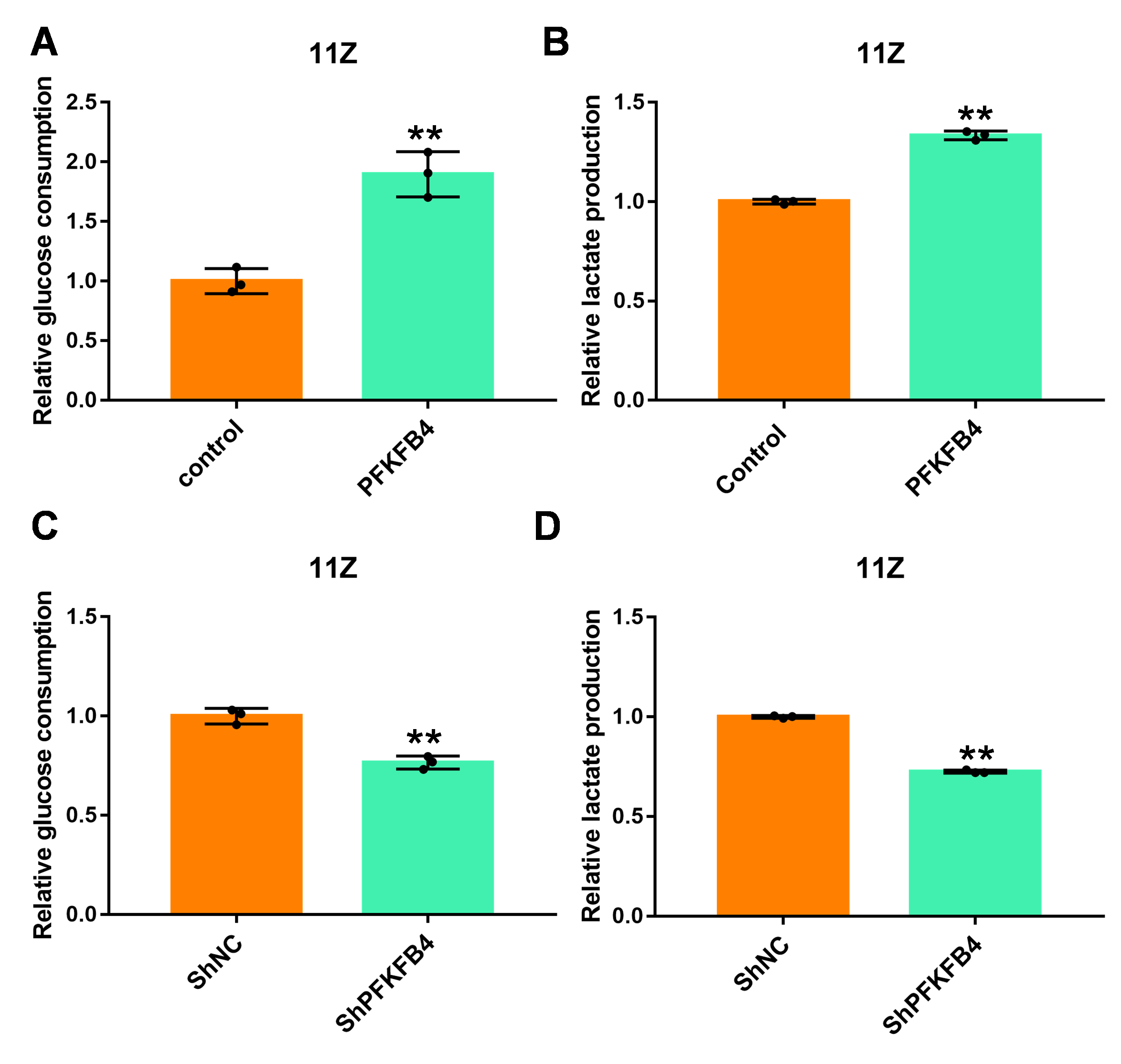

Supplement: Supplementary file 7 — Supplementary Figure S3 [file 41419_2022_5241_MOESM7_ESM.tif]

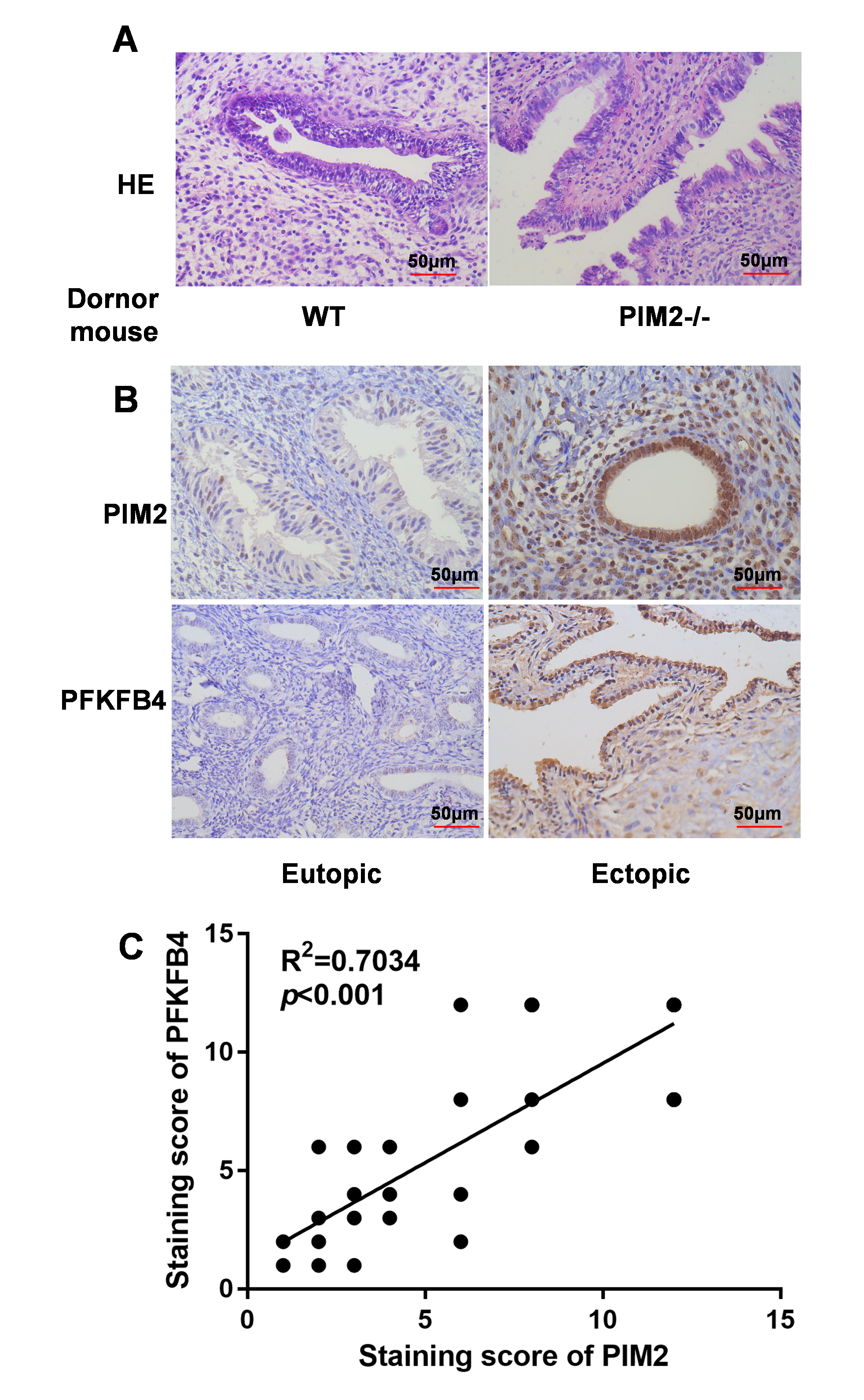

Supplement: Supplementary file 8 — Supplementary Figure S4 [file 41419_2022_5241_MOESM8_ESM.tif]
